# Performance of HADDOCK and a simple contact-based protein–ligand binding affinity predictor in the D3R Grand Challenge 2

**DOI:** 10.1007/s10822-017-0049-y

**Published:** 2017-08-22

**Authors:** Zeynep Kurkcuoglu, Panagiotis I. Koukos, Nevia Citro, Mikael E. Trellet, J. P. G. L. M. Rodrigues, Irina S. Moreira, Jorge Roel-Touris, Adrien S. J. Melquiond, Cunliang Geng, Jörg Schaarschmidt, Li C. Xue, Anna Vangone, A. M. J. J. Bonvin

**Affiliations:** 1 Bijvoet Center for Biomolecular Research, Faculty of Science - Chemistry, Utrecht University, Padualaan 8, 3584CH Utrecht, The Netherlands; 2 CNC - Center for Neuroscience and Cell Biology, FMUC, Universidade de Coimbra, Rua Larga, Polo I, 1ºandar, 3004-517 Coimbra, Portugal; 3 James H. Clark Center, Stanford University, 318 Campus Drive, S210, Stanford, CA 94305 USA

**Keywords:** D3R, Drug design data resource, Docking, Binding affinity, Ranking, Intermolecular contacts

## Abstract

We present the performance of HADDOCK, our information-driven docking software, in the second edition of the D3R Grand Challenge. In this blind experiment, participants were requested to predict the structures and binding affinities of complexes between the Farnesoid X nuclear receptor and 102 different ligands. The models obtained in Stage1 with HADDOCK and ligand-specific protocol show an average ligand RMSD of 5.1 Å from the crystal structure. Only 6/35 targets were within 2.5 Å RMSD from the reference, which prompted us to investigate the limiting factors and revise our protocol for Stage2. The choice of the receptor conformation appeared to have the strongest influence on the results. Our Stage2 models were of higher quality (13 out of 35 were within 2.5 Å), with an average RMSD of 4.1 Å. The docking protocol was applied to all 102 ligands to generate poses for binding affinity prediction. We developed a modified version of our contact-based binding affinity predictor PRODIGY, using the number of interatomic contacts classified by their type and the intermolecular electrostatic energy. This simple structure-based binding affinity predictor shows a Kendall’s Tau correlation of 0.37 in ranking the ligands (7th best out of 77 methods, 5th/25 groups). Those results were obtained from the average prediction over the top10 poses, irrespective of their similarity/correctness, underscoring the robustness of our simple predictor. This results in an enrichment factor of 2.5 compared to a random predictor for ranking ligands within the top 25%, making it a promising approach to identify lead compounds in virtual screening.

**Electronic supplementary material:**

The online version of this article (doi:10.1007/s10822-017-0049-y) contains supplementary material, which is available to authorized users.

## Introduction

Molecular docking is a widely-used tool in computer-aided drug design to model the three-dimensional (3D) structure of protein–ligand complexes, study their interactions and predict their binding affinities [1]. Integrated with data from the experimental techniques like X-ray crystallography and Nuclear Magnetic Resonance, docking has become a powerful tool in designing novel therapeutics [2]. Docking consists of two main steps: (i) exploration of protein–ligand binding poses (sampling) and (ii) identification of biologically relevant models (scoring). Both steps have their own challenges such as the flexibility of entities and the accuracy of the scoring functions. These have been reviewed elsewhere [2–4].

Our integrative, information-driven, flexible docking approach HADDOCK [5, 6] addresses this structural modeling problem by using the available experimental and bioinformatics data to drive the docking process in combination with a simple but robust scoring function for ranking. The success of HADDOCK in modeling protein–protein, protein-nucleic acid and protein–peptide complexes has been demonstrated numerous times (for a review, see [7]). HADDOCK is also consistently among the top scorers and predictors [8] in The Critical Assessment of Predicted Interactions (CAPRI) experiment [9], where participants are expected to predict the 3D structure of an unknown biomolecular complex, given the sequence or the structure of the unbound partners.

While HADDOCK has also been used in several protein–ligand docking studies [4, 10–16], no systematic benchmarking has been reported so far, making the D3R Grand Challenge 2 a perfect opportunity to assess its performance for this type of problem for which it was not originally developed. In this manuscript, we describe our strategy for predicting the binding poses of FXR ligands (Stage1), and assessing their binding affinities (Stage2), while discussing the main lessons learned from the challenge.

## Materials and methods

### Data

The target of the D3R Grand Challenge 2 is the Farnesoid X nuclear receptor (FXR), which is a nuclear hormone receptor activated by bile acids [17]. FXR is highly expressed in liver, intestines and kidneys, playing an important role in the regulation of bile acid homeostasis and cholesterol, lipid and glucose metabolisms [17–19]. Due to its involvement in various diseases including inflammatory bowel disease, colorectal cancer and type 2 diabetes, FXR agonists have emerged as potential therapeutics [17–19].

In the D3R Grand Challenge 2, the FXR dataset consists of 36 crystal structures with a resolution below 2.6 Å and binding data (IC50s) for 102 compounds, including the 36 for which a crystal structure is available (these were only made available in Stage2). These data have been provided by Roche and curated by D3R. The challenge consists of two stages, which are described below:

Stage1: The goal is to predict the poses of 35 ligands (one target is cancelled), and the affinities or rankings of all 102 compounds. The input files provided by organizers are the apo crystal structure of FXR and 2D ligands in SMILES and SD file formats.

Stage2: The participants are expected to predict the affinities or rankings of all 102 ligands with the 36 crystal structures of FXR-ligand complexes provided as additional input compared to Stage1.

### Ligand preparation

SMILES strings of FXR-ligands were converted into 3D structures using OpenEye Omega Toolkit 2.6.4 [20]. Conformers were directly generated from SMILES by Omega torsional sampling, where the maximum number of conformers per ligand was set to 100. After this step, the conformers were clustered to select representative models to be used in the docking stage. We used for this the jclust hierarchical clustering of the MMTSB tools [21], with the maximum number of clusters set to 10 and the minimum number of structures per cluster to 4. For each ligand in Stage1, an ensemble of conformations was created by selecting a representative structure from each cluster.

### Protein preparation

Docking simulations in Stage1 were run using an ensemble of 4 structures as input for the receptor. This final set of 4 receptors was selected as follows:

1. 28 Homologue structures were found in the RCSB/PDB database [22] using the “Sequence” search feature with the sequence of the apo form of FXR provided by D3R and a lower limit of 80% sequence identity. All other parameters were kept as default (Search algorithm: BLAST, Expectation value: 10, Mask low complexity: yes). We also specified that structures must contain a ligand.
2. Interface residues were extracted from all homologous structures using a 5 Å cutoff. All residues containing an atom located at 5 Å or less from the ligand were then considered as interface. The union of all these residues was taken and matched to the target sequence. The list of residues was manually curated to remove residues on the outer surface of the receptor. We then refined the residues based on their surface accessibility (SA) in the FXR apo structure (<40% backbone or sidechain SA) using NACCESS [23]. Finally, some residues with a SA below 40% were reintroduced manually (mainly residues in loops). The identified interface residues were subsequently used for clustering the receptor (see point 3 below).
3. Any structure with one or more gaps at the interface was discarded (11) leaving 18 structures (17 homologues + 1 apo) for the calculation of a pairwise backbone-RMSD after a fitting step on the interface residues using ProFit [24]. HADDOCK’s default clustering method [25] was applied on the RMSD matrix and generated 4 clusters when used with 0.5 Å threshold and a minimum cluster size of 2. It is worth noting that the apo structure was not clustered with these criteria. Two other structures (1ot7_B [26] and 3p88 [27]) were not clustered as well. Cluster representatives with the best resolution and 1ot7_B were chosen as templates. 3p88 was discarded because it was too close from a representative of cluster #2.
4. Based on 4 templates (1osv [26], 1ot7_B, 3dct [28], 3olf [29]), a new set of interface residues were computed using a 4 Å cutoff to define if a residue was interacting with the ligand or not. These residues were used as active residues in the docking runs (see Table S1 in Online Resource for the list).
5. For ensemble docking with HADDOCK, we mutated all residues diverging from the reference structure (apo form) to the respective residue with PyMOL [30]. Ensemble docking refers to the use of multiple starting conformations for one or more of the binding partners within the same docking run. All combinations of the various conformations are selected as starting point for the docking. How many times each conformation is sampled will thus depend on the number of conformation in the ensemble and the number of generated models at the rigid-body docking stage (see “Docking” below).

### Revised protocol for ligand and protein preparation in Stage2

In Stage2, 36 crystal structures for FXR1-36 protein–ligand complexes were provided by the organizers. We used those structures to revisit our docking protocol and identify the major limiting factor for our docking performance in Stage1. By docking with either bound ligand or receptor, we found that it is mainly the receptor conformation that limits our accuracy in generating near-native poses (see “Results and discussion” section). Accordingly, we identified the ligand that is most similar to FXR1-36 for targets FXR37-102 based on the Tanimoto distance calculated using fmcsR [31] and ChemmineR packages [32]. The corresponding receptor conformation was used as the protein input for all docking runs in Stage2.

As for input ligand ensemble, we followed the Stage1 protocol with an additional criterion enriching the major cluster: For the cases where less than 10 clusters were identified, remaining elements of the major cluster were additionally included in the docking ensemble, until the ensemble size reached the maximum of 10.

Access to the experimental structures of the ligands allowed us to examine the accuracy of the OMEGA generated conformers. The top panel of Fig. S1 in Online Resource provides an overview of the RMSDs of the ligand poses. The median RMSD of the generated poses for all targets was 1.9 Å, the median RMSD of the poses selected for docking for stage 1 was 2.2 Å and the median RMSD of the poses selected for stage 2 was 1.8 Å. Overall, OMEGA generated accurate—if not quite near-native—models.

### Docking

Docking was performed with the HADDOCK2.2 web server [6]. The docking protocol of HADDOCK consists of three stages: (i) rigid-body docking by energy minimization from random orientations of the starting conformations—“it0” stage, (ii) semi-flexible refinement of the interface by simulated annealing in torsion angle space—“it1” stage and (iii) short molecular dynamics refinement in explicit solvent—“water” stage. In the semi-flexible stage (it1), protein interface residues (all those within 5 Å of the ligand) and the ligand are treated as flexible. The calculations are guided by the ambiguous interaction restraints defined based on the binding pocket of the receptor (Point 2 under protein preparation above). For the D3R competition we used the buried settings of the small ligand protocol which had been benchmarked on the ASTEX dataset [33] [unpublished data]. Compared to the HADDOCK default settings, the buried binding site protocol scales the intermolecular energy terms (van der Waals and electrostatic) by a factor of 0.001 to allow penetration of the ligand into the protein binding site. This is required since the starting configurations for docking are randomly rotated and separated molecules. Accordingly, because models can contain clashes due to the scaling down of intermolecular interactions, the weight of the van der Waals energy term for scoring the initial rigid-body docking poses (it0) was set to 0.

Additionally, we fine-tuned the docking settings for Stage1 by testing on various structures of the FXR receptor bound to a plethora of ligands (namely 1osv, 1ot7, 3dct, 3hc5 [34], 3olf, 3omm [29]). Using the SMILES strings of those ligands we created ensembles of conformers as described in the “Ligand preparation” section, which we proceeded to dock against the ensemble of receptors generated during “Protein preparation” stage. The models were then compared with the bound complexes to determine the final docking settings. Based on those results, and considering the buried and rather hydrophobic nature of the binding pocket, we decided to base our selection of poses on the models obtained after the semi-flexible refinement stage (it1) of HADDOCK instead of the final, water-refined models. We increased the sampling to 10,000 and 400 poses for it0 and it1, respectively. All docking settings were left at default values except for the ones listed in Table S1 in Online Resource. The parameters and topologies for the ligands were obtained automatically by the HADDOCK server using a local version of PRODRG [35], which discards non-polar hydrogen atoms.

In both stages, two sets of restraints were provided to the server to guide the docking: (1) ambiguous interactions restraints in which the ligand and all residues in the binding pocket were defined as active to draw the ligand inside it—this was only used in it0 (50% of those restraints were randomly deleted for each docking trial); (2) unambiguous interaction restraints in which only the ligand was defined as active and the protein binding pocket as passive were used for the subsequent flexible refinement stage (it1). In this refinement phase, no energy penalty is generated if a binding pocket residue does not contact the ligand, which allows the ligand to explore the binding site. The top 5 poses from it1 stage were selected for submission.

The scoring function used for ranking the poses is the standard HADDOCK score for the flexible refinement (it1) which is defined as:

1 $${\text{HADDOCK score}}~=~1.0 \times {{\text{E}}_{{\text{vdW}}}}+1.0 \times {{\text{E}}_{{\text{elec}}}}\quad+1.0 \times {{\text{E}}_{{\text{desol}}}} - 0.01 \times {\text{BSA}}$$

where BSA is the buried surface area in Å^2^, E_desol_ an empirical desolvation energy term [36]. The intermolecular energies are calculated using the OPLS united atom force field parameters [37] for non-bonded atoms, using a 8.5 Å cut-off with a shifting function for the electrostatic energy and switching function between 6.5 and 8.5 Å for the van der Waals energy. For the electrostatics energy, a dielectric constant of 10 is used.

### Binding affinity prediction

For Stage1 of the challenge, we used the HADDOCK score to rank the affinities of 102 compounds. For Stage2, we developed both a ligand-based and a structure-based binding affinity predictor, which are described below.

### Ligand-based binding affinity predictor

We designed a target-specific ligand based binding affinity predictor, based on the assumption that similar ligands binding to the same protein should have similar binding affinities. From the database BindingDB [38], we retrieved 229 ligands that bind to the FXR protein with reported experimental IC50 data. We calculated the ligand similarity using Atom Pair (AP) and Maximum Common Substructure (MCS) measurements, as implemented in ChemmineR and fmcsR packages [31, 32]. For this, we computed the pairwise similarity matrix among the training data (i.e., the 229 ligands). This matrix was used to train a Support Vector Regression (SVR) model using LibSVM software (version 3.21) [39]. During the training process, we transformed IC50 data into ln(IC50). We evaluated the SVR predictor on the training data using 10 repeats of 5-fold cross-validation. The AP metric outperformed the MCS metric (Table 1). We, therefore, in the subsequent analysis used AP to train our predictor. The binding affinity of the D3R ligands was then calculated using our predictor with the similarity matrix between the 102 D3R ligands and the training data (the 229 ligands from BindingDB).

**Table 1:** Comparison of the prediction performance of atom-pair and maximum common substructure predictors on the training dataset using 10 repeats of 5-fold cross-validation

	Atom-pair	Maximum common substructure
Kendall’s Tau	0.52 ± 0.01	0.50 ± 0.01
Pearson’s correlation coefficient	0.70 ± 0.01	0.68 ± 0.02

### Structure-based binding affinity predictor

Recently, we have introduced a residue–residue contact-based method for the prediction of the binding affinity in protein–protein complexes [40], implemented in the webserver PRODIGY (PROtein binDIng enerGY prediction) [41, 42]. This simple structural-based approach has led to one of the best performing predictors so far reported on a large and heterogeneous set of data [43, 44], with Pearson’s Correlation of 0.73 between the predicted and the experimental values and a root mean-squared error of 1.89 kcal mol^−1^.

For Stage2 of this D3R challenge we designed an adapted version of our contact-based prediction for protein–ligand complexes. From the 2P2I database [16], we retrieved 200 protein–ligand complexes with experimentally measured Ki (inhibition constant) and available crystal structure. Ki values were converted to free energy (ΔG) by applying the equation ΔG = RTln(Ki), in which R is the gas constant and T the temperature. For each entry, we ran the HADDOCK refinement protocol in order to collect the intermolecular energy terms reported in Eq. 1. This consists of the final refinement stage of HADDOCK without any initial perturbation of the starting structures. We then calculated the number of atomic contacts (ACs) within the distance threshold of 10.5 Å (this cutoff was optimized to obtain the best correlation). We further classified the ACs according to the atom involved in the interaction (C = Carbon, O = Oxygen, N = Nitrogen, X = All other atoms). We used this combination of structural- and energy-based terms to train a multiple linear regression model with R [45] performing 4-fold cross validation. We applied Akaike’s Information Criterion (AIC) stepwise selection method implemented in R to avoid overfitting and identify the significant features. The resulting binding affinity predictor ΔG_score_ model for ranking the targets based is shown in Eq. 2:

2 $${{{\Delta}\text{G}}_{{\text{score}}}}=0.343794 \times {{\text{E}}_{{\text{elec}}}} - 0.037597 \times {\text{A}}{{\text{C}}_{{\text{CC}}}}+0.138738 \times {\text{A}}{{\text{C}}_{{\text{NN}}}}+0.160043 \times {\text{A}}{{\text{C}}_{{\text{OO}}}} - 3.088861 \times {\text{A}}{{\text{C}}_{{\text{XX}}}}+187.011384$$

where AC_CC_, AC_NN_, AC_OO_ and AC_XX_ are the ACs between Carbon–Carbon, Nitrogen–Nitrogen, Oxygen–Oxygen and between all other atoms and polar hydrogens, respectively. E_elec_ is the electrostatic energy calculated through the HADDOCK refinement protocol.

For each of the top 10 it1 poses from the docking runs we calculated the ΔG_score_ and took the average. We finally ranked the ligands according to the predicted values of our averaged ranking-score.

## Results and discussion

### Binding pose predictions

**Fig. 1:**
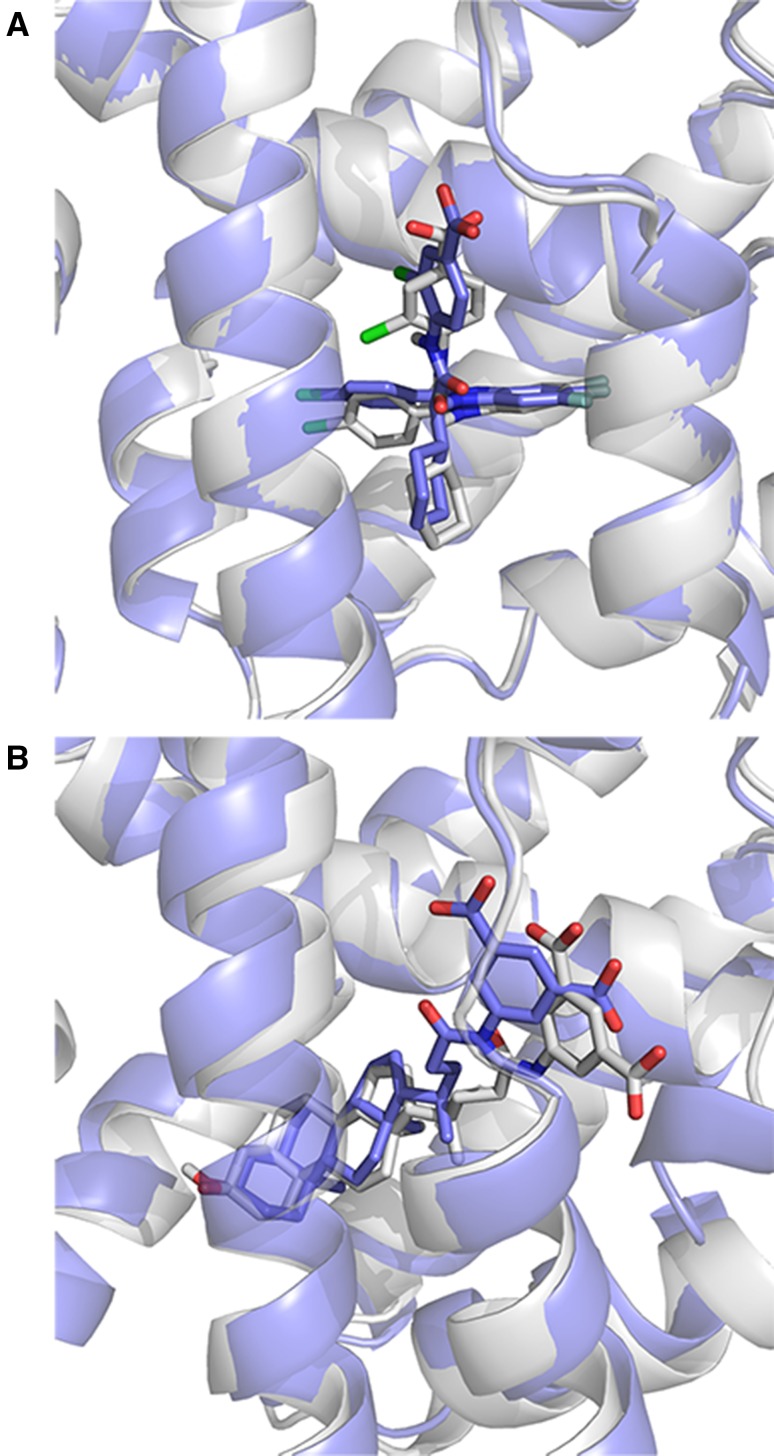
Examples of successfully predicted ligand poses in Stage1 for (**a**) FXR-27 (**b**) FXR-34 with a l-RMSD of 1.27 and 1.94 Å, respectively. The receptor conformations are shown as cartoon and the ligands as *stick* representation. The reference crystal structure is *colored grey* and the model as slate

**Fig. 2:**
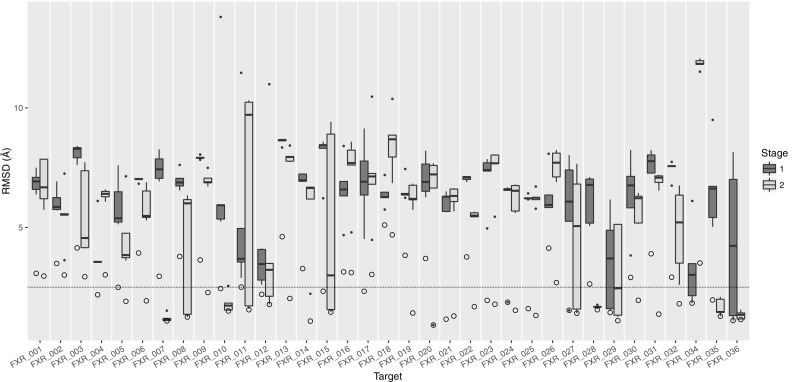
Comparison of the l-RMSDs of the top5 scoring poses between stages 1 and 2. l-RMSD values of the top5 poses are drawn as *boxplots* with the values of Stage1 *colored dark gray* and those of Stage2 *light gray*. The *black line* in the *middle of the boxes* corresponds to the median, the *lower* and *upper* hinges correspond to the 25th and 75th percentile respectively, the *whiskers* extend to no longer than 1.5 times the IQR from the hinge. Any point beyond that range is considered an outlier and drawn as a *filled black point*. The *circles* correspond to the overall minimum l-RMSD obtained in it1 for that target. In the cases where the *circle* overlaps with an outlier or a *boxplot*, the minimum l-RMSD structure is part of the top5 scoring poses. The *dotted line* represents the l-RMSD cutoff of 2.5 Å. The number of successful predictions increases from 6/35 in Stage1 to 13/35 in Stage2

**Fig. 3:**
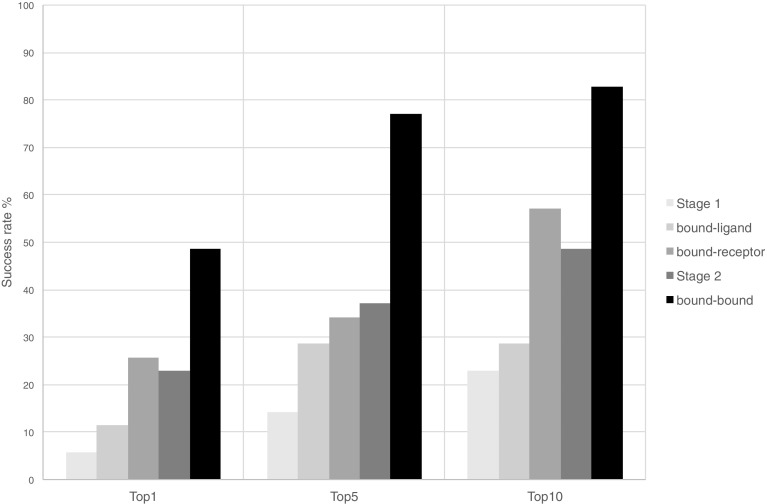
Successful prediction (l-RMSD < 2.5 Å) rates for top1, top5 and top10 in different docking runs for 35 targets. Bound-ligand docking refers to runs with bound ligand conformer and the ensemble of receptors used in Stage1. Bound-receptor is the one with bound receptor and the ensemble of ligands used in Stage1. Finally, bound–bound is the bound receptor-bound ligand docking runs

Following the protocol described in Methods, we submitted 5 binding poses per target in Stage1. Two of the successfully predicted cases are shown in Fig. 1, where the ligand RMSD (l-RMSD, defined as the RMSD of the ligand heavy atoms after fitting on receptor backbone) is less than 2.5 Å. The performance per target in the prediction phase is indicated in Fig. 2 (dark grey box plots) for our submitted five poses. We have at least one model within 2.5 Å of the bound state in 6 out of 35 targets with an average l-RMSD of 5.1 Å for all targets. This rather low performance encouraged us to revisit the ligand and protein preparation protocols, as described in “Revised protocol” section. In particular, we investigated whether conformational changes/sampling is the limiting factor (Fig. 3). Our docking performance in Stage1 is compared to that using either the bound ligand, bound receptor or both. Our performance reaches 83% success rate for bound–bound docking. The largest improvement compared to Stage1 is obtained if the bound conformation of the receptor is used. Moreover, revisiting the ligand sampling also increased the docking success from 14 to 20% for top5 (data not shown). This prompted us to select for Stage2 the receptor conformation containing the most similar ligand to the ligand to be docked (see “Material and methods”) and a resampled ensemble of ligand conformations. The resulting improvement can be easily observed in Fig. 2 (light grey box plots), where the average l-RMSD is reduced to 4.1 Å and 13 out of 35 targets are within the 2.5 Å cut-off. We can also clearly see that there is plenty of room for optimizing our scoring function since in most cases we did generate reasonably good predictions (shown as circles) in the pool of 400 refined models, but these did not make it in the top5.

**Fig. 4:**
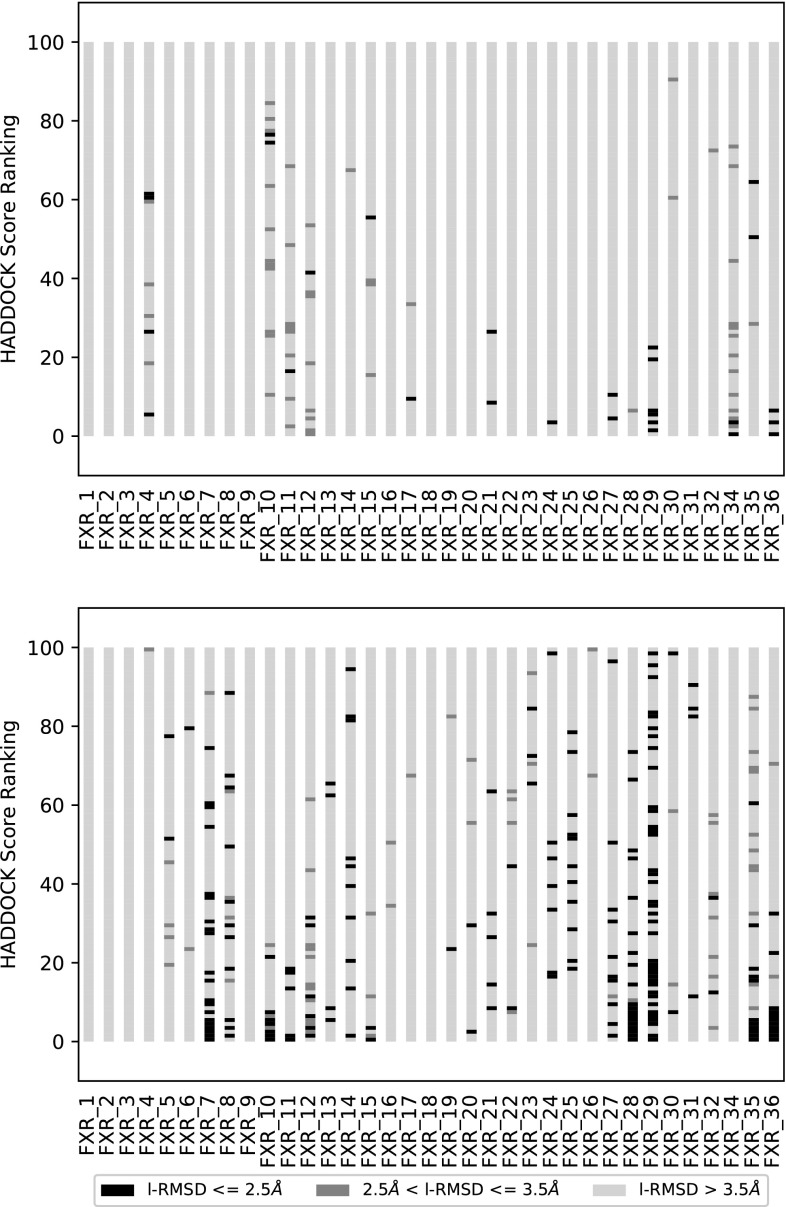
Comparison of the top100 models for the protocols used for stages 1 and 2. Each *bar* corresponds to structures belonging to runs for the indicated target. The *coloring of the bars* separates the structures in 3 classes. Structures *colored black* have a l-RMSD smaller than 2.5 Å, structures *colored dark gray* have a l-RMSD between 2.5 and 3.5 Å and structures with a l-RMSD of greater than 3.5 Å are *colored light gray*. The top-ranked structures are the ones close to zero on the y-axis

Additionally, we investigated whether the revised protocol improves the sampling. Figure 4 compares Stage1 and Stage2 binding poses, where the y-axis reflects the ranking of the top 100 structures at the end of it1 for each target, with higher ranked structures being close to zero. The coloring of the bars depends on the l-RMSD of the model to the bound complex, with darker shades corresponding to lower l-RMSD values. As is evident from Fig. 4, the revised protocol dramatically improves the sampling as low l-RMSD structures are identified and tend to be ranked higher.

We should also note that the ligand parameters were obtained automatically by the HADDOCK server using PRODRG—the only currently supported option on the server—with its known limitations. Especially the accuracy of the charge assignment by PRODRG can be questioned [46]. In a previous study on the prediction of the binding affinity of protein–protein interaction inhibitors [16], we have compared PRODRG and ACPYPE [47] for ligand parameter generation showing that the HADDOCK score calculated with the two parametrizations scheme are correlated (R^2^ = 0.73). While the van der Waals and desolvation energies are essentially identical, the electrostatic energies differ substantially (R^2^ = 0.33), which might well affect the quality of our docking poses.

### Binding affinity

#### Ligand-based binding affinity prediction

**Fig. 5:**
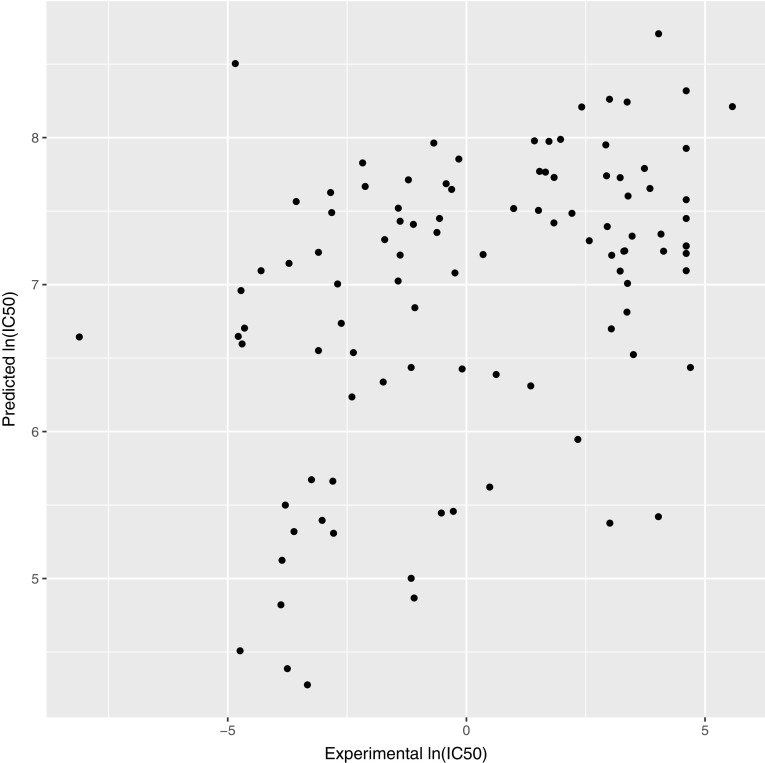
Comparison of predicted ln(IC50) with experimental ln(IC50) using our ligand-based binding affinity predictor

A Support Vector Regression model based on ligand similarity using Atom Pair (see “Material and methods”) was used for ligand-based prediction of the binding affinities. The Kendall’s Tau between the ranking of the experimental and our predicted binding affinities is 0.27, which is the third best performance out of five participants. The correlation between the two sets can be visualized in Fig. 5.

Although this method does not perform as well as our structure-based predictor (see below) it has as major advantage that it does not require a structural model and is therefore extremely fast.

#### Structure-based binding affinity prediction

**Fig. 6:**
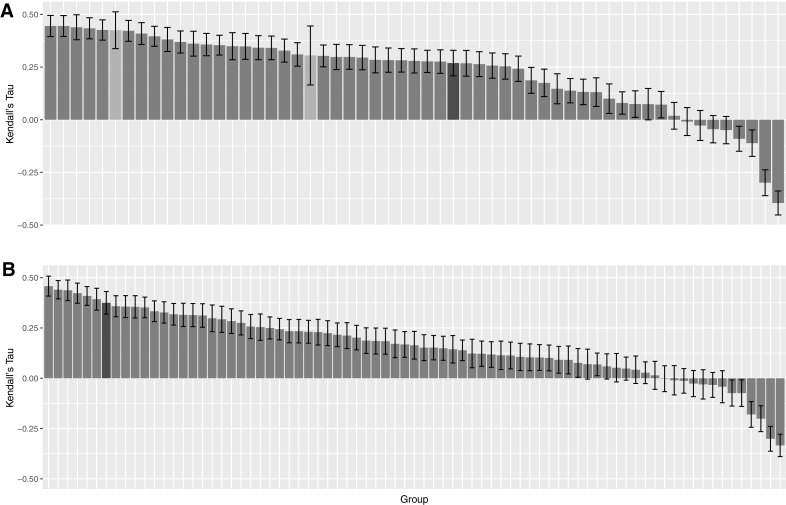
Ranking of binding affinity correlation per group for stages 1 and 2. The *top panel* reports the results of Stage1 and the *bottom* one of Stage2. *Bars colored light gray* correspond to groups which did not provide submissions for all targets. The *bars colored dark gray* correspond to the HADDOCK group submission

The correlation scores (Kendall’s Tau) of the binding affinity rankings calculated for stages 1 and 2, for all groups are summarized in Fig. 6. We clearly performed better in Stage2 with a correlation of 0.37 against 0.27 in Stage1, where we used only HADDOCK scores for ranking. In terms of Pearson’s Correlation coefficient between the predicted scores and the experimental binding affinity, our prediction performance improved from 0.40 in Stage1 to 0.51 in Stage2 with the structure-based predictor (see Online Resource—Fig. S2). Interestingly, averaging the ΔG_score_ over the top10 models resulted in a correlation of 0.37 while using only the top scoring model yielded 0.28. Considering that our top10 poses are rather heterogeneous in their conformations, our binding affinity predictor seems rather robust and not too sensitive to the exact conformation of the ligand. Further investigations are needed to dissect those results and investigate the impact of energetics and the quality of the models on the ranking performance.

**Fig. 7:**
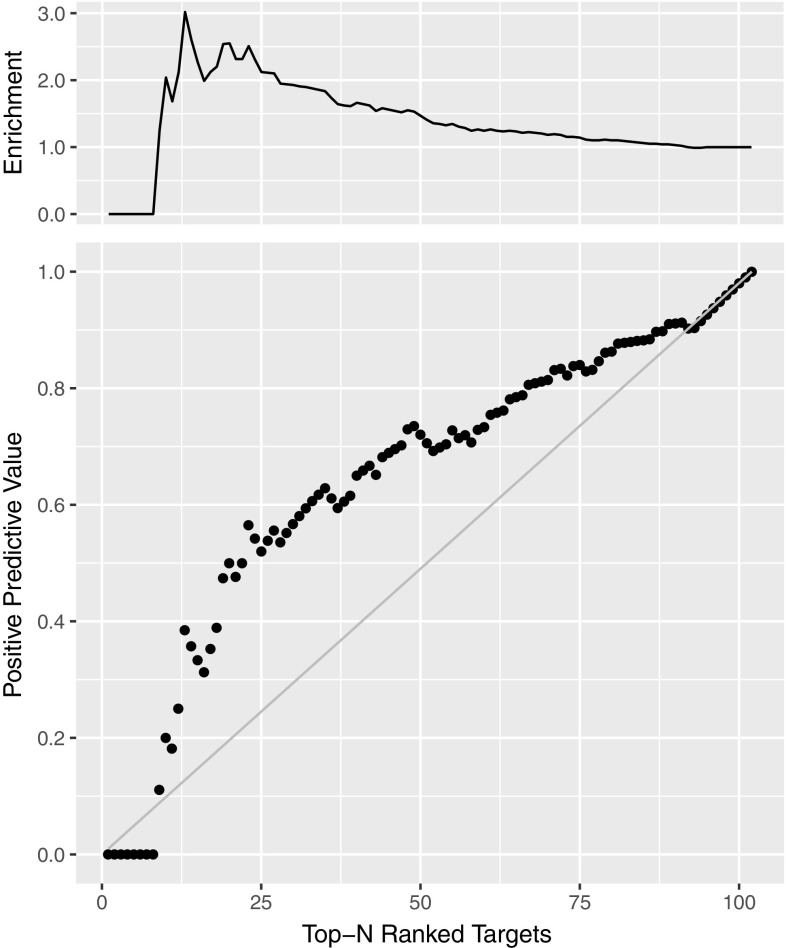
Positive predictive value (*bottom*) and enrichment factor (*top*) for 102 targets, using structure-based binding affinity predictor. Taking top 20–25% is associated with 2.5 enrichment factor

We also investigated the potential of our ranking predictor for identification of lead compounds. We defined as true positive the targets which are within the top N ranked compounds of both the predicted and experimental binding affinity rankings (N: 1,2…,102). Then, we calculated the positive predictive value (PPV), which is equal to the number of true positives divided by the number of predicted positives (top N ranked targets according to BA predictor). We plotted PPV as a function of N together with the diagonal which represents a random prediction (RP) (Fig. 7). We also report the enrichment factor (PPV/RP) on the top of Fig. 7. This analysis indicates that our predictor reaches a 2.5-fold improvement in correct identification of effective ligands in the top 20–25% compared to random.

## Conclusions

Our participation in the D3R Grand Challenge 2 was an opportunity to evaluate and revisit our docking and ranking protocols. Our pose prediction performance in Stage1 was far from optimum, which led us to investigate the effect of ligand/protein conformer selection on the docked model quality. We identified the conformation of the receptor as main limiting factor, which led us to select receptor conformers for Stage2 based on ligand similarity, which significantly improved our pose prediction performance. This, together with a biasing of the major cluster for ligand conformers as explained in ‘Revised protocol’ increased our overall prediction success.

As for ranking in Stage2, we developed two different BA predictors: A ligand-based one and structure-based one. Our ligand-based predictor is computationally efficient since it does not require any 3D structural model for training. However, it does not perform as well as our structure-based predictor (Kendall’s tau is 0.27 and 0.37 for ligand and structure-based, respectively). Using the structure-based predictor, which considers the number and type of interatomic contacts, for affinity ranking dramatically improved our overall performance for binding affinity prediction, with our ranking compared to the other submitted methods improving from 32nd/57 for Stage1 to 7th/77 for Stage2 (and if only considering a single submission per group per category, from 18th/27 (Stage1) to 5th/25 (Stage2) among all groups participating to the challenge).

As final observation, it is worth noting that our ranking was based on the average score calculated over the top 10 poses (which are heterogeneous in most cases, particularly with respect to the ligand orientation in the binding pocket—see Fig. 2). This averaging yielded better predictions than only using the top1 (Kendall’s tau 0.37 and 0.28 for top10 and top1, respectively). This simple contact-based predictor seems to show promise as virtual screening tool to select a fraction of effective ligands, yielding an enrichment factor of about 2.5 for the top 25% of compounds compared to a random selection.

## Electronic supplementary material

Below is the link to the electronic supplementary material.

Supplementary material 1 (PDF 1930 KB)
