# Supplementary material for: Performance of HADDOCK and a simple contact-based protein–ligand binding affinity predictor in the D3R Grand Challenge 2
Source: J Comput Aided Mol Des. 2017 Aug 22;32(1):175–85. doi: 10.1007/s10822-017-0049-y (PMC5767195; doi:10.1007/s10822-017-0049-y)
Supplement: Supplementary file 1 — Supplementary material 1 (PDF 1930 KB) [file 10822_2017_49_MOESM1_ESM.pdf]

Supplementary Information

Performance of HADDOCK and a simple contact-based protein-ligand binding affinity predictor in the D3R Grand Challenge 2

Zeynep Kurkcuoglu[1*], Panagiotis I. Koukos[1*], Nevia Citro[1], Mikael E. Trellet[1], J.P.G.L.M. Rodrigues[3], Irina S. Moreira[1,2], Jorge Roel-Touris[1], Adrien S.J. Melquiond[1], Cunliang Geng[1], Jörg Schaarschmidt[1], Li C. Xue[1], Anna Vangone[1], A.M.J.J. Bonvin[1]

[1]Bijvoet Center for Biomolecular Research, Faculty of Science - Chemistry, Utrecht University, Padualaan 8, Utrecht, 3584CH, the Netherlands
[2]CNC - Center for Neuroscience and Cell Biology; Rua Larga, FMUC, Polo I, 1ºandar, Universidade de Coimbra, 3004-517, Coimbra, Portugal.
[3]James H. Clark Center, Stanford University, 318 Campus Drive, S210, Stanford, CA 94305, USA

# To whom correspondence should be addressed: a.m.j.j.bonvin@uu.nl, phone: +31 30 2533859
*Joint first authors

Table S1. HADDOCK parameters used for dockings

Parameter	Setting
It0 sampling (rigid body docking)	10,000
It1 sampling (semi-flexible simulated annealing)	400
Delenph	False
Inter_rigid	0.001
Tadinit2_t	500
Tadfinal2_t	50
Tadinit3_t	500
Tadfinal3_t	50
Initiosteps	0
Cool1_steps	0
W_vdw_0	0
Protein interface residue list	269,273,274,277,287,288,291,292,294, 295,298,332,333,335,336,339,340,346,347, 352,356,359,361,365,369,370,373,451,454,458,469,473
amb = ExtStageConstants (firstit = 0, lastit = 0,)	

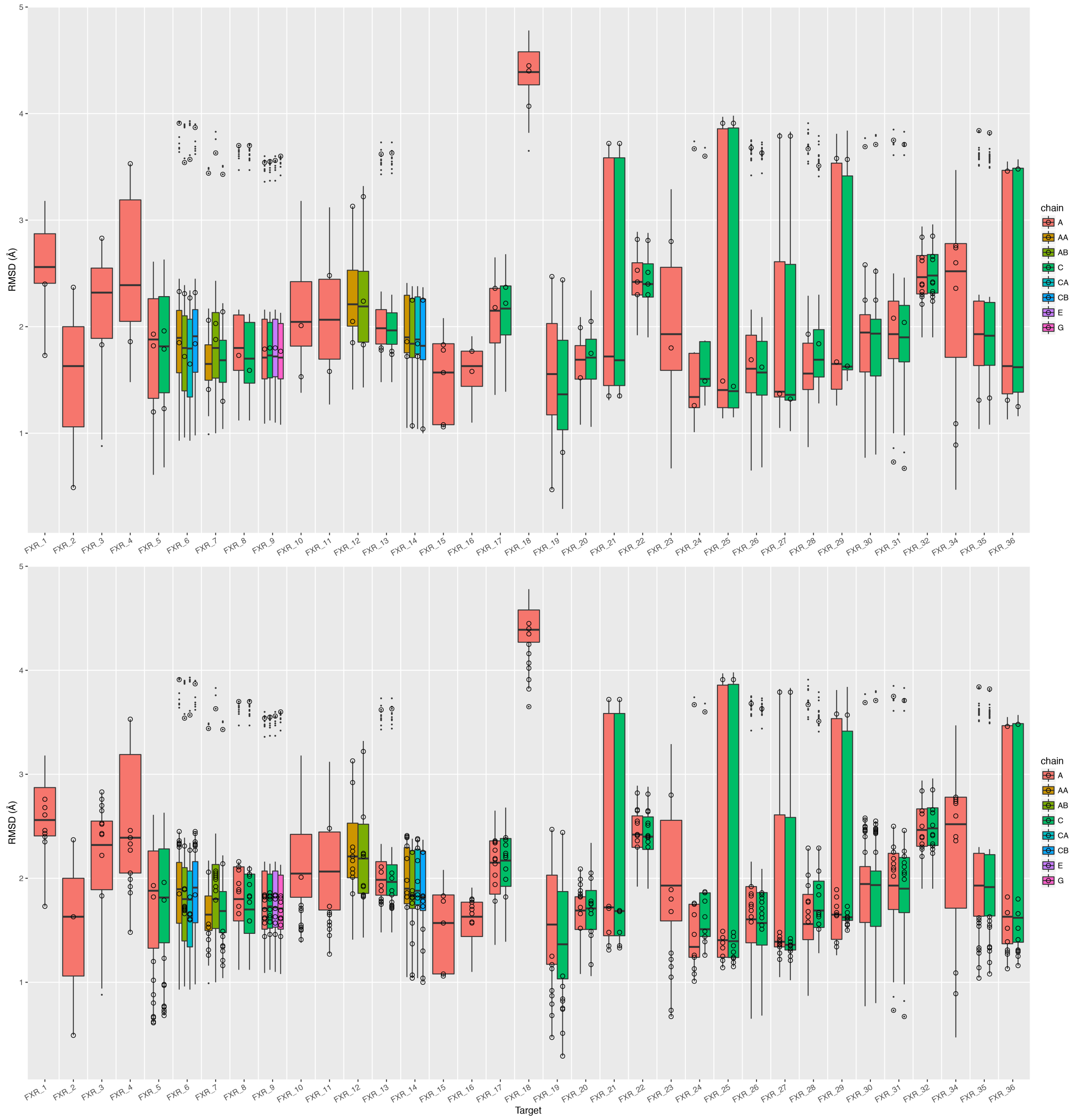

Figure S1: RMSD values of the OMEGA generated ligand conformers against the reference structures. The boxplots are colored according to the chain ID of the reference chain used for the calculations. Circles indicate the RMSD values of the poses that were selected for docking. The top panel corresponds to the conformer selection for stage 1 and the bottom one to the conformer selection for stage 2.

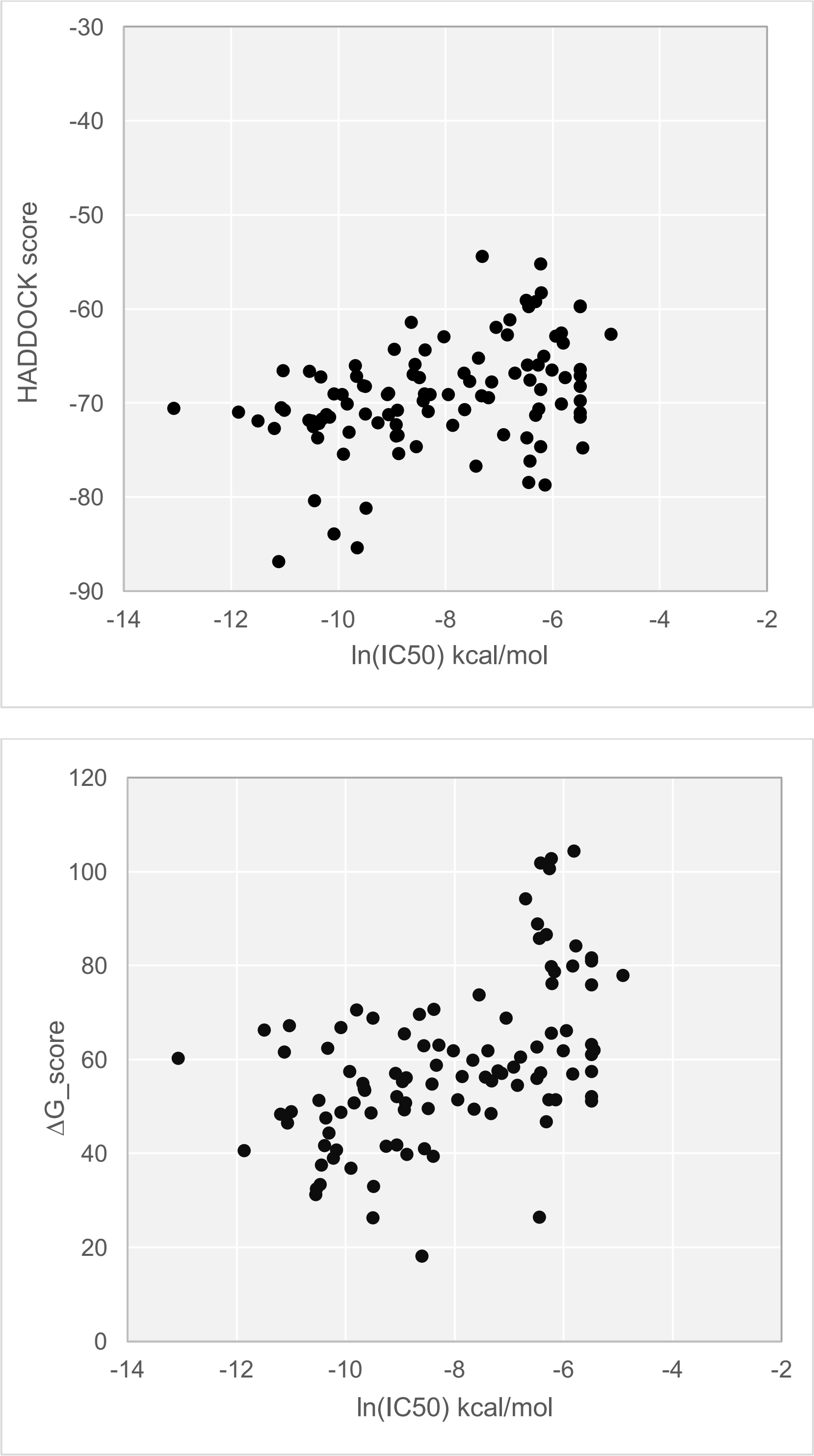

Figure S2: Scatter plot between the HADDOCK score for Stage 1 (top panel) and ΔGscore for Stage 2 (bottom panel) versus the experimental binding affinities reported as ln(IC50). The corresponding Pearson's correlation coefficients are 0.40 and 0.51 for Stage 1 and Stage 2, respectively. The ΔG_scores have been calculated with our structure-based binding affinity predictor (see Eq. 2 in the main text), averaged over the top10 best models refined with the refinement interface of the HADDOCK2.2 web server.
